# Health and work: regional health reporting on employees in Germany

**DOI:** 10.1007/s43999-023-00028-4

**Published:** 2023-09-13

**Authors:** Matthias Richter, Karin Kliner, Dirk Rennert

**Affiliations:** Federal Association of Company Health Insurance Funds (BKK Dachverband), Berlin, Germany

**Keywords:** Health insurance, Employees, Incapacity to work, Inpatient care, Outpatient care, Drug prescription

## Abstract

The vast majority of people work a large part of their live, therefore work inevitably has a major impact on health as well. In Germany, great efforts are made to improve health and well-being in the occupational context. To support this, the evaluation of health data especially from employees is one the main scopes in health reporting of the Federal Association of Company Health Insurance Funds (BKK Dachverband). These results are made available to the interested public via various publication channels, including interactive online diagrams. Data from the areas of incapacity to work, outpatient and inpatient care as well as drug prescriptions are applied. Due to the in large parts identically structured representations, comparisons across health care system sectors are also possible. The aim of this article is to describe the provided regional health care data of employees with focus on the interactive data provision, to show by example particularities in single sources and across sources. From this the impact of work as well as workplace health promotion on the health of employees and, in a broader sense, the quality of health care in Germany can be derived.

## Introduction

Health is an important value with a great importance for the individual as well as for society. In addition, companies have a very own interest in healthy and productive employees. Accordingly in Germany various state as well as economic institutions research on the connection between work and health. Their main aim is the maintenance as well as the improvement of individual health in the work context. Occupational safety is a legal obligation, health promotion has also been established for years. Health insurance companies are among these institutions, of which it is the company health insurance funds (BKK) constitute a direct link between companies, employees and health. In this regard, they can look back on a long tradition: The first German company health insurance fund was founded in 1717 in the Blaufarbenwerk Pfannenstiel in Aue, Saxony [1]. Until the twentieth century, the BKKs were exclusively responsible for single companies as health insurance providers. With the introduction of free choice of health insurance for all insured persons through the Health Structure Act [2], the majority of the BKKs opened up from 1996 to be eligible for all persons liable or entitled to insurance. Within the statutory health insurance (SHI), the BKK is one of six different types of health insurance funds schemes that has evolved historically [3]. Overall, about 15% of all insured persons in the German SHI-system are insured within a BKK [4].

The Federal Association of Company Health Insurance Funds (BKK Dachverband), which was founded in Berlin in 2013, acts as the representative and advocacy for the BKKs at the national level. It professionally and politically represents the interests of its members, these are currently (as of 2023) 66 company health insurance funds (BKK) and 4 regional associations. The BKK Dachverband is a registered association with voluntarily membership and is financed by its participating company health insurance funds. Around 9 million people are insured through these members in total, about half of whom are employees subject to social insurance contributions.

The BKK Dachverband also performs health reporting tasks, with the annual Health Report (BKK Gesundheitsreport) as one of the most important publications in this context. This report, which has been published since 1976 (previously until 2013 by the BKK Bundesverband, the mandatory organization of the BKKs. Thereafter the BKK Dachverband continued this work), is probably the oldest publication of such health insurance statistics. Initially, only statistics on incapacity to work were reported, but now it also includes data from outpatient and inpatient care as well as drug prescriptions. Since several years, the editors set a specific focus subject, which is deepened with more detailed analytics as well as external contributions from science, politics and practical experts.

The data sources of the BKK Gesundheitsreport (which are described in more detail in the following section) are also made available to the interested public in digital form. In particular, the interactive statistics in form of diagrams for interested users on the homepage of the BKK Dachverband ( www.bkk-dachverband.de ) should be pointed out. All of these data sources are primarily used in the economic context: Health insurance funds as well as companies and even science and politics use it for insights in the health situation of employees, specific particularities in the health behavior and the use of the health care system. Insights into the prevalence of diseases, their treatment and correlations with socio-demographic, regional and work-related factors can be derived and with this prevention and health promotion in the context of §20a and §20b of Book V of the German Social Code (SGB V) could be promoted.

The aim of this article is to describe the regional health care data and its impact on the quality of health care respectively workplace health promotion in Germany by using the population of all employees insured at a BKK. For this, it is described by the example of cardiovascular diseases how noticeable information could be distracted from each dataset by itself and by comparing these sources.

## The underlying data of health reporting

The four primary data sources that are used in health reporting, which were mentioned before, are described below. It applies to all data sources, that they represent the administrative prevalence of illnesses. That means that primarily not the prevalence of a certain illness could be derived from the health reporting data, but they show how many people make use of the health system because of that illness and what treatment is used for the cure. For this, all data are collected from administrative instances (especially medical services, health insurance funds and employers) and not from the insured persons themselves. Due to the legal basis for recording the use of medical services, it can be assumed that almost all events are recorded. It includes the whole time period; it is not based on fixed day data collection. Nevertheless all health data pass through quality checks (check for completeness, duplicates, correct coding etc.). Further details about the data sources, calculation bases and key figures are described in the chapter “Methodical Notes” of the BKK Gesundheitsreport [5].

### Incapacity to work data

The data on incapacity to work are mainly based on data supplied by the BKKs as part of the compilation of the official statistics. Incapacity to work is ascertained by a physician, employees get a medical report with diagnoses as well as start and end date about their incapacity to work for submission to employers and health insurance companies. Hence, the days of incapacity to work are calendar days, not only workdays.

All cases of incapacity to work, the days of incapacity to work and the resulting duration of all cases completed in the specified reporting year are included in the analyses. This in turn results in the corresponding sick leave rate (percentage of calendar days in the period under review that each employee is incapacitated to work on average due to illness). These key figures on incapacity for work include both periods with continued payment of wages as well as those with sickness benefits. These statistics also take into account cases and days of incapacity to work that occur in the context of commuting and work accidents and in the course of medical rehabilitation. Exactly one main or first-mentioned diagnosis (coded according to ICD-10-GM [6]) is assigned to each case of sick leave according to the documented sick note.

In addition to the statistics for the individual reporting years, monthly statistics on sick leave are also collected. These BKK monthly statistics enable to observe variations in data during the year and show current developments even before the end of a reporting year. The BKK monthly statistics deviate from the annual statistics due to their collection and analysis methodology; a comparison of the monthly data with the annual data is therefore only possible to a limited extent.

### Inpatient care data

The hospital data used, just like the data on incapacity for work, are based on the data supplied by the BKK as part of the compilation of the official statistics. All inpatient care cases completed in the reporting year are included in the analyses and the number is determined as calendar days, as well as the resulting case duration (days per case). In addition, it should be noted that only inpatient or day-care hospital cases are taken into account. Hospital days are counted including admission and discharge days. All cases are assigned to ICD diagnoses [6] according to the discharge diagnosis documented per case.

### Outpatient care data

The data on outpatient care are based on billing information from service providers. For outpatient diagnoses, all individual case records from the reporting year with a valid ICD diagnosis [6] are included in the analyses. Thereby only confirmed diagnoses are included. In contrast to the data on incapacity to work or inpatient care, where only the respective main or discharge diagnosis is used for the analyses, in outpatient care data all confirmed diagnoses per insured person and reporting year are taken into account. In this health care sector, the percentage of persons affected with a corresponding diagnosis is reported as a key figure. The proportion of those affected in outpatient care is determined based on the occurrence of at least one diagnosis in the reporting period. Therefore, the reported dates for outpatient care are not cumulative.

### Drug prescription data

The drug prescription data are also based on billing information from service providers. All reimbursable individual prescriptions of pharmacy-only drugs whose prescription date is in the respective reporting year are taken into account. The reported key figures are based exclusively on prescriptions from the outpatient sector. The key figures reported are the individual prescriptions, the defined daily doses (DDD) and the percentage of insured persons with at least one prescription. For this last key figure, the same methodology is used as for the percentage of persons affected in outpatient care, which are also not cumulative, whereas the DDD are cumulative. The classification of the prescribed pharmacological agents is based on the Anatomical-Therapeutic-Chemical Classification (ATC; [7]).

### Health services in these sectors

These four data sources reflect the respective care reality of the health care sector from which they originate. In inpatient care, for example, there are mainly serious illnesses, which only affect a relatively small proportion of the population. Accordingly, inpatient treatment is rarely used (in 2021 this only affected just under 11% of those insured with BKK), so the number of cases in this health care sector is correspondingly significantly lower than in the others. The clearest contrast to this is outpatient care: Normally more than 90% of the insured persons have been treated there at least once a year. This reveals a very broad picture of the situation in health care: In addition to types of illness that can severely impair those affected (and are therefore relevant in other service areas), other diagnoses can also be found in outpatient care that relate to check-ups, prevention etc. These diagnoses are mainly relevant in the outpatient setting, but are only slightly relevant in other sectors of the health system. For example, eye or skin diseases are rarely so serious that they lead to incapacity to work or inpatient treatment. In addition, drug treatment does not necessarily result from this either. However, drug treatments are also relatively common; around 70% of BKK insured persons received at least one prescription in 2021. Incapacity to work, on the other hand, only refers to the part of the insured persons who are generally employable with an entitlement to sickness benefit. Of these, almost 50% have been medically diagnosed as incapable to work at least once a year.

### BKK-insured employees in comparison to the employed population in Germany

Finally we want to compare the population of employees insured in a company health insurance funds (BKK-insured employees) to the population of all employees subject to social insurance contributions in terms of age, sex and place of residence [8, 9] (for further details see [10]).

In 2021 (and in all reported years before) the majority of employees in Germany are men (BKK: 54.7%; Overall: 53.7%). Around two thirds of all employees are between 25 and 54 years old (BKK: 68.1%; Overall: 67.6%), around one in ten is younger than 25 years (BKK: 9.1%; Overall: 9.8%). Despite the increase in the standard retirement age to 67, the proportion of employees over the age of 65 is low (BKK: 0.9%; Overall: 1.3%).

**Fig. 1:**
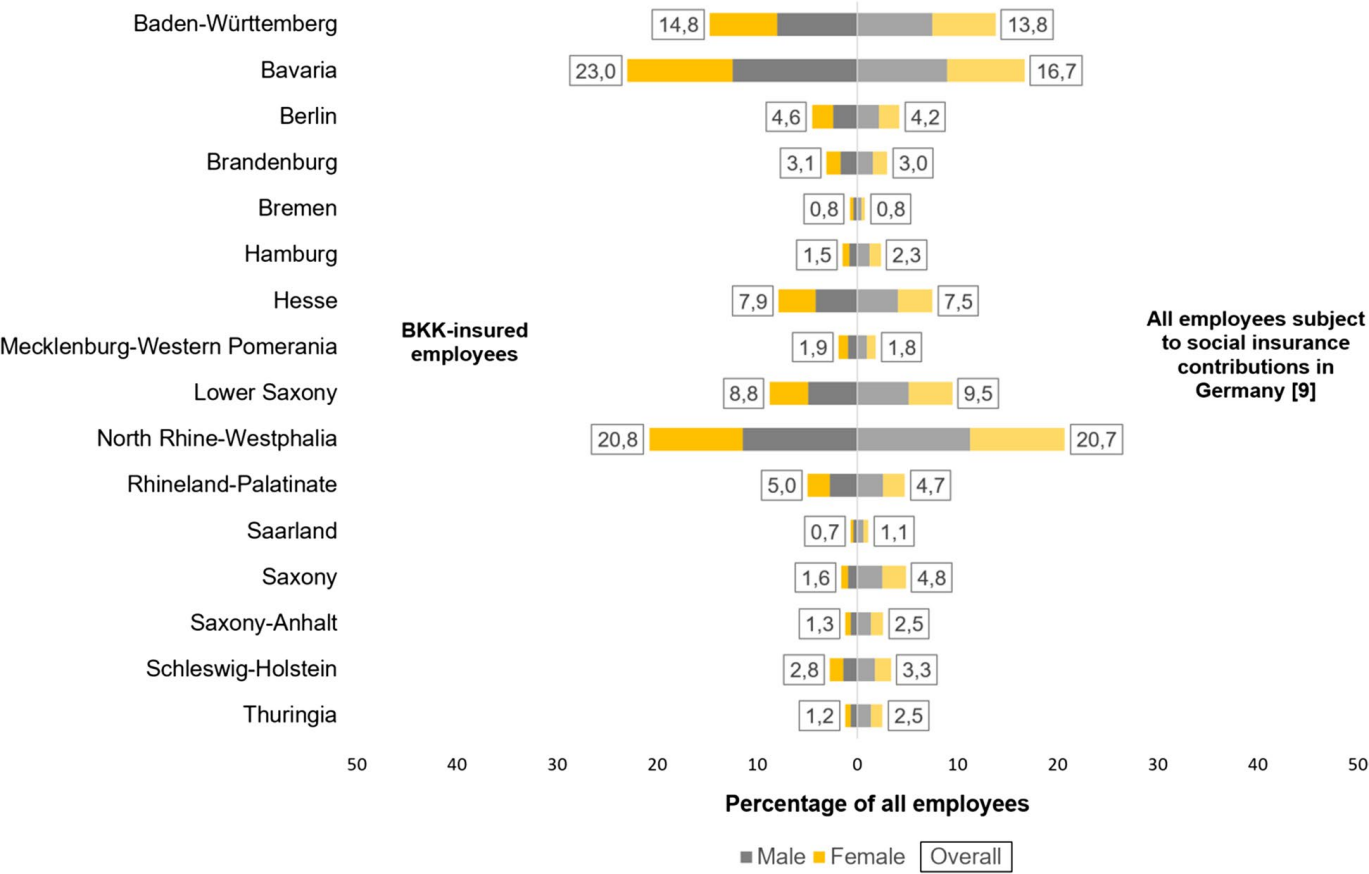
BKK-insured employees in comparison to the employed population in Germany for the reporting year 2021

As shown in Fig. 1, most of the BKK-insured employees (together 58.6%) as well as most of the employees subject to social insurance contributions (together 51.2%) live in Baden-Württemberg, Bavaria and North Rhine-Westphalia. The employees in Bavaria in particular are disproportionately often members of a BKK (BKK: 23.0%; Overall: 16.7%). For most other federal states, the proportion of BKK-insured employees roughly corresponds to the proportion of all employees subject to social insurance contributions in Germany. In the eastern federal states, on the other hand, BKK-insured employees tend to make up a smaller proportion compared to the total number of employees. In summary, despite marginal deviations, it can be stated that the population of the BKK-insured employees essentially corresponds to that of all employees subject to social insurance contributions in Germany.

Since the employees represent the largest group within all insured persons, it is obvious that there are no major differences for all BKK insured persons compared to the total population of the statutory health insured people. They are quite similar in terms of their sociodemographic characteristics:

- 49.7% of all BKK-insured are female. Within all SHI-insured the proportion of women is 51.8%.
- Also only minor differences in the age groups can be found. The largest group consists of the 25 to 54 year olds, followed by those younger than 25 years. The greatest difference to all those with statutory insurance is still among those insured over the age of 65 (BKK: 18.5%; SHI: 22.4%).

Further details about are described in the chapter “Sociodemographic Characteristics” of the BKK Gesundheitsreport [5].

## Publicly available regional health data

**Fig. 2:**
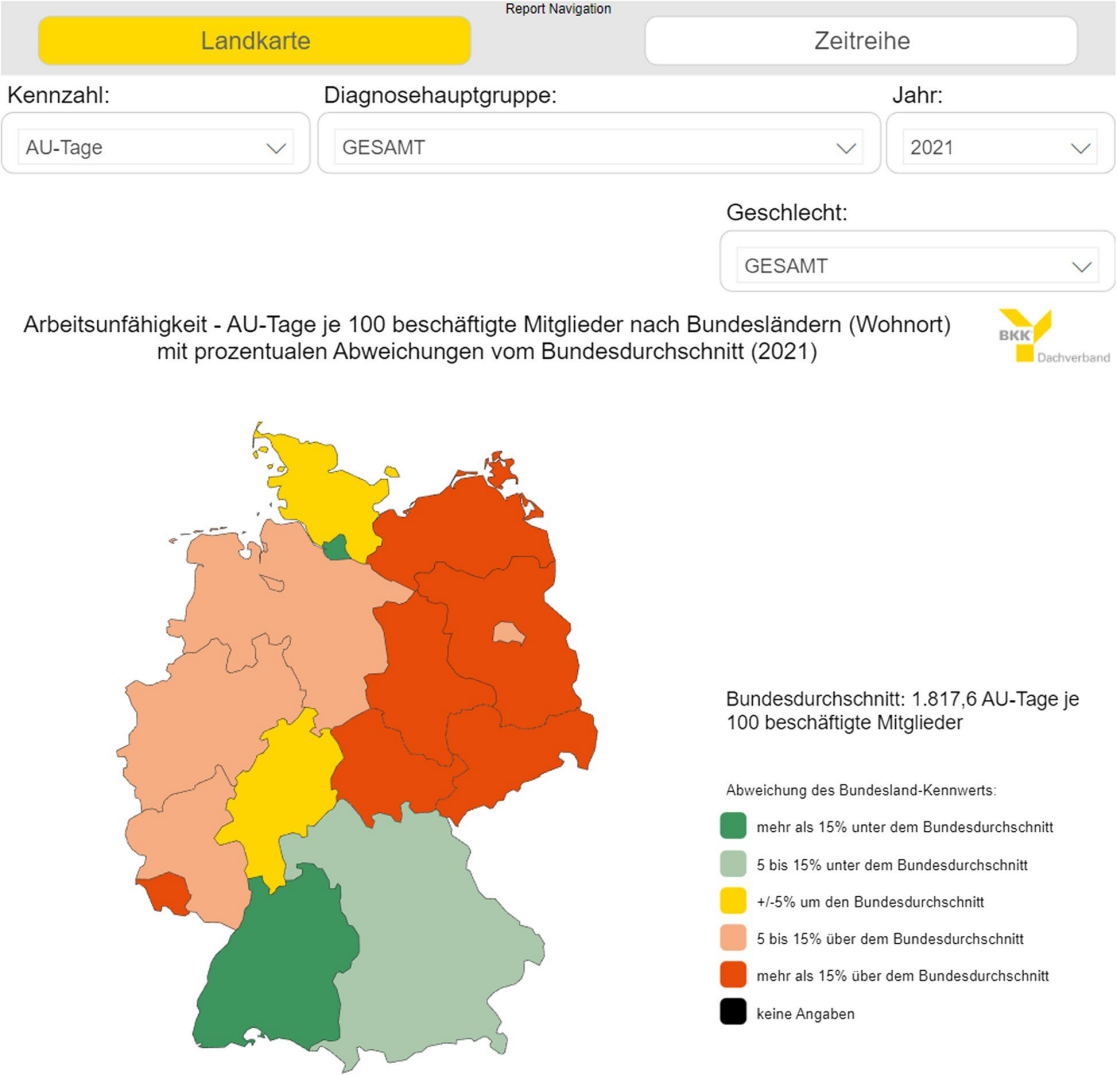
Screenshot of the interactive map of employee sick leave days by federal state (place of residence) for the reporting year 2021. Source: https://www.bkk-dachverband.de/statistik/kennzahlen-zum-bkk-gesundheitsreport/arbeitsunfaehigkeit

Data from these four health reporting sources outlined above are made available to the interested public. In particular, large amounts of data were transferred into interactive diagrams. Currently (as of July 2023), three interactive diagrams can be found on the homepage of the BKK Dachverband for each of the four health care sectors, which differ in terms of the following variables:

- A map with regional data by federal state (according to the main residence of the insured person) (as an example a screenshot of the diagram for incapacity for work data from the webpage is shown in Fig. 2),
- a diagram with differentiations according to economic sections and divisions (according to the classification of economic activities: WZ 2008 [11]) as well as
- a diagram with differentiations according to occupational segments and main groups (according to the classification of occupations: KldB 2010 [12]).

In these interactive diagrams (they can be found on the following webpage: https://www.bkk-dachverband.de/statistik/kennzahlen-zum-bkk-gesundheitsreport), the specific data for the reporting years from 2016 can be called up, while the monthly statistics on sick leaves always include the last three reporting years. In addition, a differentiation option is offered according to sex, types of illness or ATC groups for the drug prescriptions. Users can configure the maps and diagrams individually by these filters, whereby the initial selection is immediately displayed in the interactive diagram.

### Exemplary data observation: cardiovascular diseases

Below, using the example of diseases of the circulatory system (ICD-10 codes I00-I99), it is shown which content can be accessed in the publicly provided interactive diagrams.

#### Cardiovascular diseases as a reason for incapacity to work

**Fig. 3:**
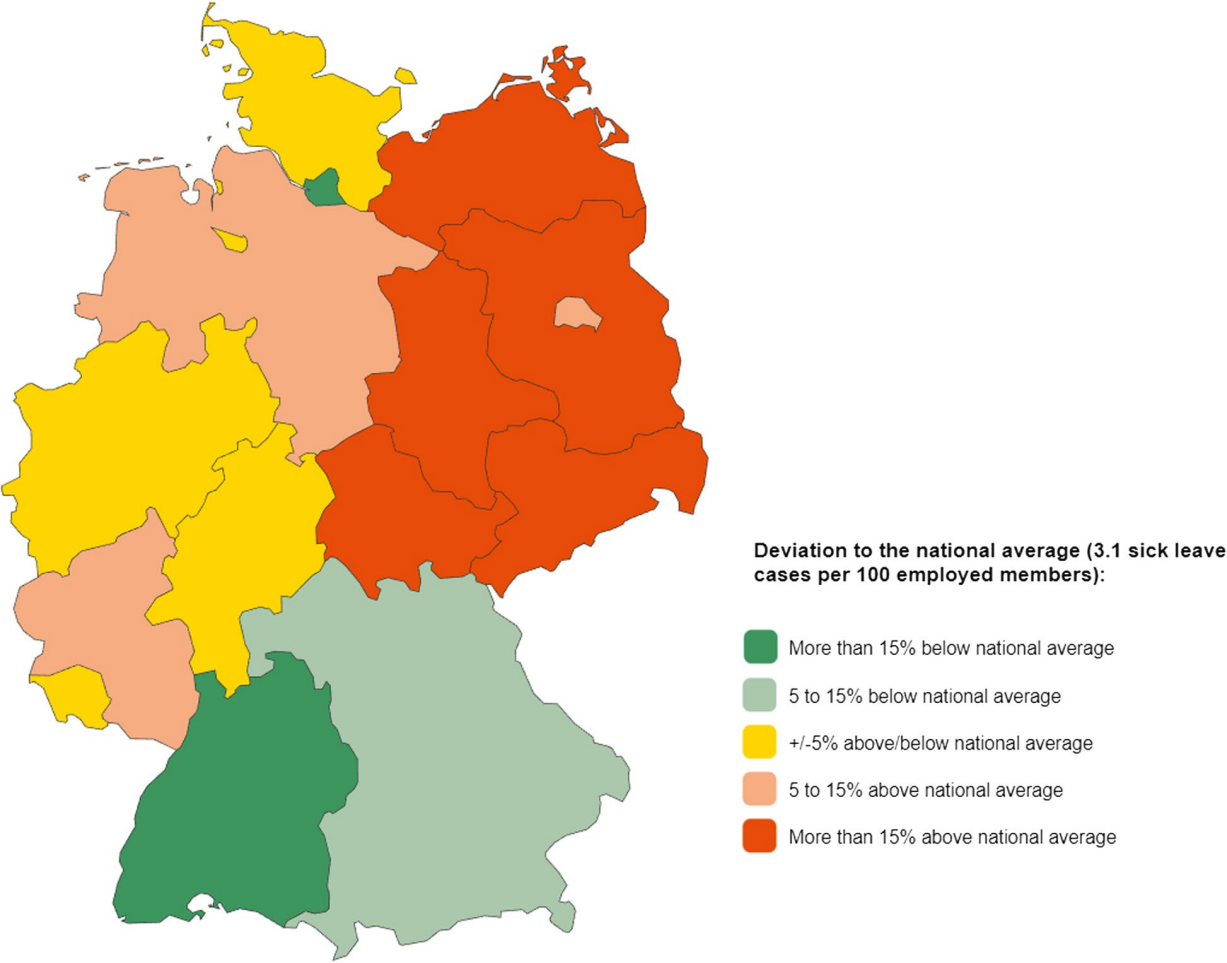
Map of employee sick leave cases due to diseases of the circulatory system by federal state (place of residence) for the reporting year 2021. Source in German, translated in English: https://www.bkk-dachverband.de/statistik/kennzahlen-zum-bkk-gesundheitsreport/arbeitsunfaehigkeit

Two point six percent of all cases and 4.1% of all days of incapacity to work are related to cardiovascular diseases. This indicates that these diseases do not play a major role in this sector. Nevertheless, as Fig. 3 shows, there are bigger regional differences, as cardiovascular diseases are an above-average reason for cases of incapacity to work particularly in eastern Germany. With the exception of Berlin (the small federal state in the northeast of Germany), all eastern German federal states have a more than 15% higher number of cases (dark red coloring of the state areas) than the national average (3.1 cases of incapacity to work per 100 employed members). The highest average number of cases is in Saxony-Anhalt with 5.3 cases of incapacity per 100 employed members. In contrast, this indicator is less than half as high in Hamburg (2.2 cases of incapacity to work per 100 employed members), followed by Baden-Württemberg (2.6 cases of incapacity to work per 100 employed members), both of which have a lower number of cases by more than 15% (dark green coloring of the areas of both federal states). This unequal regional distribution is due to various factors. Age differences also play a role (the average age in the eastern federal states is more than 2 years higher than the national average [13]), since the effects of wear on the cardiovascular system are related to age. However, other factors (including lack of exercise, alcohol consumption, obesity, diabetes) are known to occur more frequently in the eastern federal states [14].

#### Cardiovascular diseases in outpatient care

**Fig. 4:**
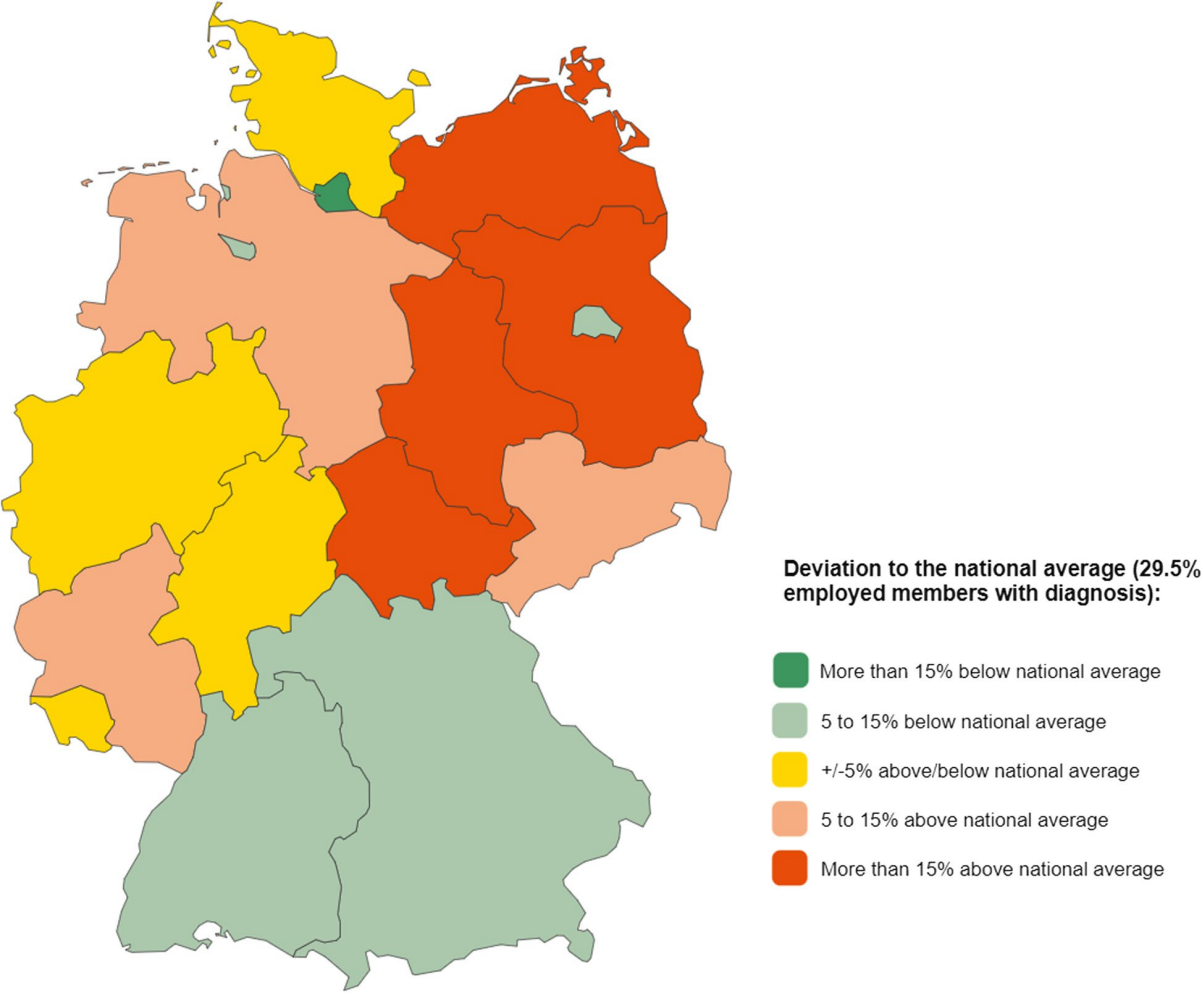
Map of the proportion of employees treated on an outpatient basis with diseases of the circulatory system by federal state (place of residence) for the reporting year 2021. Source in German, translated in English: https://www.bkk-dachverband.de/statistik/kennzahlen-zum-bkk-gesundheitsreport/ambulante-versorgung

The fact that wear effects play a major role in cardiovascular diseases can also be observed very well based on outpatient care data for all insured people. The number of people who are treated as outpatients for this type of disease increases rapidly with age: From the age of 55, more than half of the insured are usually affected, from the age of 70 it is even more than 80% [15]. For comparisons between the data sources, it is possible to look at the employed members separately in the interactive diagrams for the areas of outpatient and inpatient care as well as drug prescriptions. As Fig. 4 shows, a national average of 29.5% of the employed members received outpatient treatment for cardiovascular diseases in 2021. The regional differences by federal states in outpatient care are relatively similar to the incapacity for work described above. The most obvious difference between these health care sectors can be seen in Berlin: although there is an above-average number of cases of incapacity to work here, the proportion of employees who have been treated as outpatients for such illnesses is in contrast below average.

#### Cardiovascular diseases in inpatient care

**Fig. 5:**
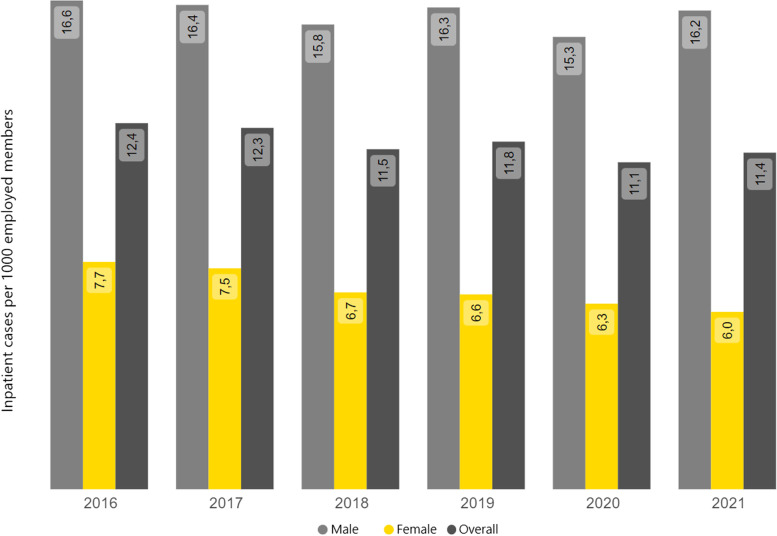
Inpatient care cases of employees living in Berlin (place of residence) due to diseases of the circulatory system in a time comparison (2016—2021). Source in German, translated in English: https://www.bkk-dachverband.de/statistik/kennzahlen-zum-bkk-gesundheitsreport/stationaere-versorgung

Diseases of the cardiovascular system are the most common reason for hospitalization in the general insured population: In 2021, 26.1 cases of inpatient care per 1000 insured persons were treated for this diagnostic group in hospital, which means that 15.0% of all inpatient treatment cases are attributable for this type of disease alone. As expected, the number of cases among employed members is lower. In the same reported year, 11.1 inpatient cases per 1000 employees were treated in a hospital for cardiovascular diseases. The regional analysis, however, shows slight differences in some federal states. For example, the number of inpatient treatment cases due to cardiovascular diseases is above average in North Rhine-Westphalia, while in Saxony it is slightly below average. In Berlin, on the other hand, this parameter is also average. As Fig. 5 also shows, the number of inpatient treatment cases for the group of male employees is twice as high as for female employees. This applies to the number of inpatient cases presented here as an example for Berlin, but also relates to the national average. In Berlin, however, there are more cases for male employees than the national average, while for women in Berlin there are fewer. Furthermore, the longitudinal representation shows that this gender difference remains stable across all reporting years. This longitudinal representation also clearly shows the influence of the coronavirus pandemic phase. The key figures fell by around -14% from 2019 to 2020. These are, among other things, the effects of various regulations (including postponement/suspension of planned treatments) that were made during the pandemic in order to minimize the risk of infection and to maintain sufficient treatment capacities [16].

A comparison of the four data areas also reveals gender differences. Men are more often incapacitated to work due to cardiovascular diseases than women are. They are also more often prescribed cardiovascular drugs and are more often treated as inpatients because of this type of disease. Women, on the other hand, are slightly more likely to receive outpatient treatment due to cardiovascular diseases than men are. In addition to BKK statistics, statistics on causes of death show that women die more frequently due to cardiovascular diseases than men do, but also at an older age [17].

#### Prescriptions of cardiovascular drugs

**Fig. 6:**
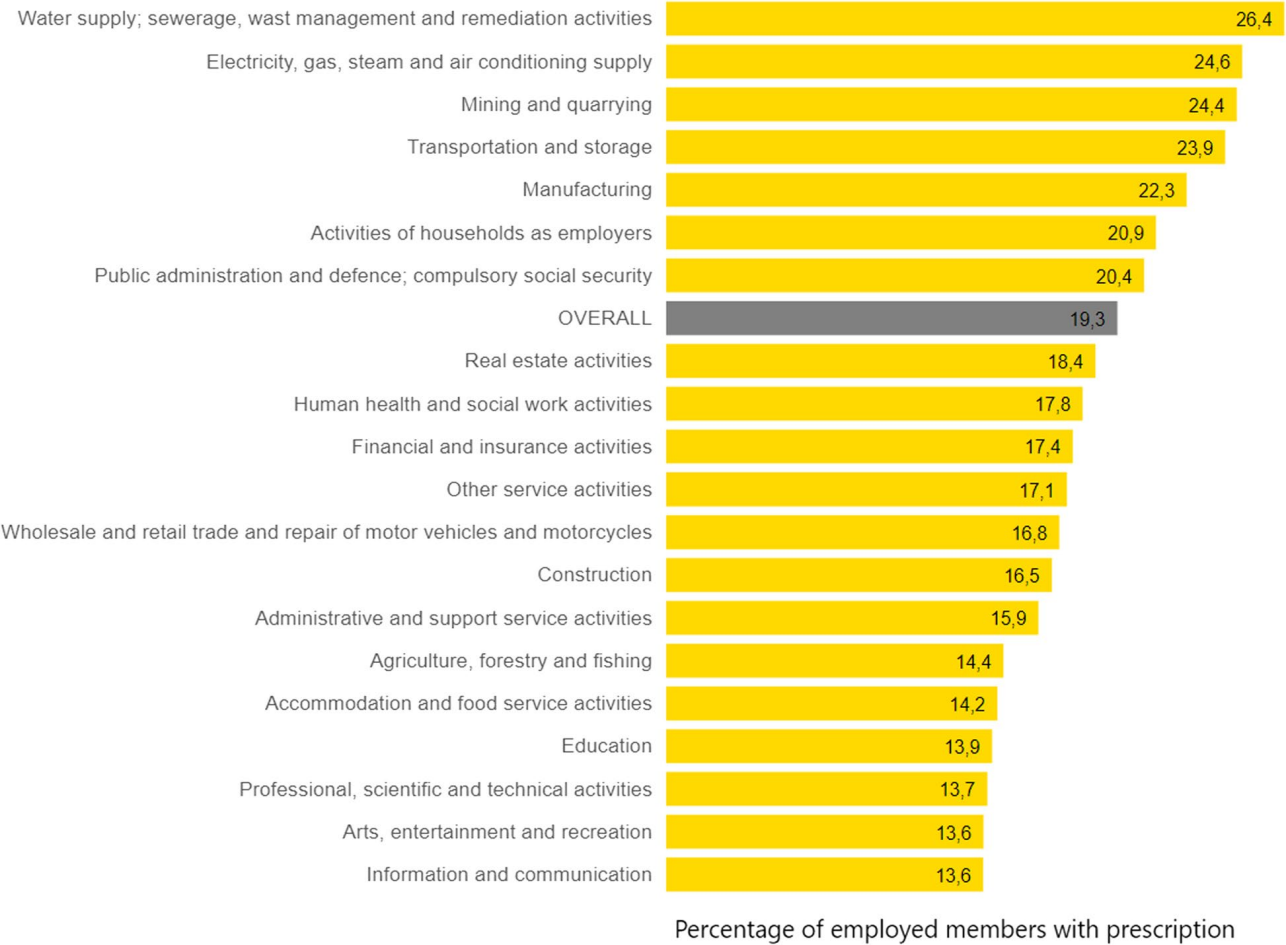
Comparison of the proportion of employees with a prescription for drugs for the cardiovascular system by economic group for the reporting year 2021. Source in German, translated in English: https://www.bkk-dachverband.de/statistik/kennzahlen-zum-bkk-gesundheitsreport/arzneimittelverordnungen

As is to be expected according to the large proportion of patients in outpatient care, cardiovascular diseases also play an important role in the drug prescription process. For the general insured population, more than every fourth individual prescription and almost half of all defined daily doses are attributable to this main anatomical group alone. In 2021, 27.1% of all insured persons received at least one prescription; this share is lower for employed members at 19.3%. If the regional differences are also considered here, the variance within the federal states is similar to that in incapacity to work (highest percentage in Saxony-Anhalt, lowest percentage in Hamburg). Here, too, only Berlin shows a difference: The proportion of employees with a prescription for the cardiovascular system is only slightly above the average in this federal state. On the other hand, clear differences can be found for other variables. As Fig. 6 shows, the proportions of those with at least one prescription for a cardiovascular drug vary depending on the economic group in which the employed members work. For example, employees in water supply, sewage and waste disposal have the highest percentage with 26.4%, while employees in the information and communication sector have a percentage that is almost half as high to this at 13.6%.

#### Coherence to other data sources

As already described, the population of the BKK-insured employees essentially corresponds to that of all employees subject to social insurance contributions in Germany and their use of health care services in terms of age, gender and place of residence (as well as other sociodemographic variables not described here [10]). Moreover, the collection of health related data by medical staff as well as the collection of sociodemographic variables based on official documentations is also reliable. For further comparisons to other data sources, it must be considered that they may differ in terms of the included populations, the survey method or the used key figures. Nevertheless, other sources confirm the patterns we find in our data regarding the health situation in Germany. With regard to cardiovascular diseases, several correspondences can be found. Other sources show similar key figures for different variables and proportions of disease groups in the different health care sectors, as well as a similar variation over time. For instance, these cardiovascular diseases play the same minor role in the official statistics for incapacity to work, nearly identical proportions of all sick leave cases and days are shown there; moreover, the official statistics also verify a peak for these key figures in age groups between 55 and 65 years [18]. Regional differences between federal states are confirmed by sick leave analyses of other health insurance funds [19]. For the three other health care sectors, comparisons are mainly possible with the overall population of SHI-insured people respectively to the complete German population, because samples of employees are used only very rarely for these sectors. For this we compare with all BKK-insured people (data for them are also available online), within this group employees are the largest subgroup. Empirical studies [14, 20] show similar regional differences with above-average prevalence in the eastern federal states, as well as very similar patterns concerning age groups and sex. Evaluations of outpatient medical care data for cardiovascular diseases (especially hypertension) [21, 22] as well as mortality statistics [23] also reveal the individual east–west-gradient already described. This is also confirmed for inpatient care by other health insurance funds data [24]. SHI-based statistics on drug prescriptions also show that, as demonstrated in the BKK data, drugs with effect on the cardiovascular system account for the by far the largest proportion of prescription quantities, they also confirm regional differences as we found in the BKK-data [25].

## Conclusion

As this article shows, health reporting by the BKK Dachverband is a valid source for extensive amounts of data from different health care sectors, which allows detailed analyzes and cross-sector comparisons. Therefore, the health reporting with its several publications (especially with the long-established BKK Gesundheitsreport) is highly valued by stakeholders in politics, economy, science, media and health care. Health insurance funds as well as companies itself use BKK publications and the underlying data for health related purposes like benchmarking in company specific health reports and workplace health promotion. In addition, cooperation projects with social insurance partners (other health insurance companies, the German statutory accident insurance and/or the German pension insurance) are also supplied with data for the health reporting by the BKK Dachverband (e.g. [26–29]). Findings from the BKK health reporting are cited in political debates (e.g. [30–32]) as well as in a draft law [33], scientific works (e.g. [34, 35]) as well as daily media (e.g. [36, 37]). Particularly the monthly data on sick leave rates is used to monitor the health of employees in Germany, especially regarding faster spreading illnesses like infections or respiratory diseases. During the coronavirus pandemic extra analyzes helped to evaluate the effects of government containment actions.

All these data give interesting insights in the health of employees and its relation to the specific particularities in the economic sections or occupations they work in. As the working world changes, several challenges lay ahead: The economy gets more global, new technologies transform complete business areas. That changes the way people work. At the same time, the demographic change progresses, the average age of employees further increases, they have more chronic diseases, etc. [38]. Because of this, it gets even more important to gain more insights in the mutual influence between health and work. Health data from health insurance funds can be an important source for this. In addition, health insurance funds can use this directly to derive tailor-made data-driven programs for workplace health promotion and make them available to the companies.

In order to meet these future requirements, the BKK publications as well as the provision of health data in general in interactive and digital form will be constantly evolved. The dovetailing of the various other digital communication channels that are already being used for publications should be further promoted. In addition, the interactive representation and data provision in particular are to be expanded even further in the future. It will not only be given a larger database by adding further reporting years, but further variables will also be added. For example, further regional details, differentiations according to age groups and other groups of insured persons as well as age- and sex-standardized key figures are conceivable.
